# Design of an Integrated Acceptance Framework for Older Users and eHealth: Influential Factor Analysis

**DOI:** 10.2196/31920

**Published:** 2022-01-28

**Authors:** Jiyeon Yu, Angelica de Antonio, Elena Villalba-Mora

**Affiliations:** 1 Research Group on Human-Computer Interaction and Advanced Interactive Systems Madrid HCI Lab Universidad Politécnica de Madrid Madrid Spain; 2 Centre for Biomedical Technology (CTB) Universidad Politécnica de Madrid Madrid Spain; 3 Centro de Investigación Biomédica en Red en Bioingeniería, Biomateriales y Nanomedicina (CIBER-BBN) Madrid Spain

**Keywords:** eHealth, older people, older user, health technology, acceptance factors, adoption, acceptance framework, systematic review, thematic analysis, influential factor analysis, mobile phone

## Abstract

**Background:**

eHealth and telehealth play a crucial role in assisting older adults who visit hospitals frequently or who live in nursing homes and can benefit from staying at home while being cared for. Adapting to new technologies can be difficult for older people. Thus, to better apply these technologies to older adults’ lives, many studies have analyzed the acceptance factors for this particular population. However, there is not yet a consensual framework that can be used in further development and to search for solutions.

**Objective:**

This paper aims to present an integrated acceptance framework (IAF) for older users’ acceptance of eHealth based on 43 studies selected through a systematic review.

**Methods:**

We conducted a 4-step study. First, through a systematic review in the field of eHealth from 2010 to 2020, the acceptance factors and basic data for analysis were extracted. Second, we conducted a thematic analysis to group the factors into themes to propose an integrated framework for acceptance. Third, we defined a metric to evaluate the impact of the factors addressed in the studies. Finally, the differences among the important IAF factors were analyzed according to the participants’ health conditions, verification time, and year.

**Results:**

Through a systematic review, 731 studies were found in 5 major databases, resulting in 43 (5.9%) selected studies using the PRISMA (Preferred Reporting Item for Systematic Reviews and Meta-Analyses) methodology. First, the research methods and acceptance factors for eHealth were compared and analyzed, extracting a total of 105 acceptance factors, which were grouped later, resulting in an IAF. A total of 5 dimensions (ie, personal, user–technology relational, technological, service-related, and environmental) emerged, with a total of 23 factors. In addition, we assessed the quality of evidence and then conducted a stratification analysis to reveal the more appropriate factors depending on the health condition and assessment time. Finally, we assessed the factors and dimensions that have recently become more important.

**Conclusions:**

The result of this investigation is a framework for conducting research on eHealth acceptance. To elaborately analyze the impact of the factors of the proposed framework, the criteria for evaluating the evidence from the studies that have the extracted factors are presented. Through this process, the impact of each factor in the IAF has been presented, in addition to the framework proposal. Moreover, a meta-analysis of the current status of research is presented, highlighting the areas where specific measures are needed to facilitate eHealth acceptance.

## Introduction

### Background

The world’s population is aging, and this phenomenon will affect the health care system for older people in the future, and we need to be prepared [1]. During the COVID-19 pandemic, it has been revealed that older people are an especially risky group, and public health authorities have advised them to stay safely at home [2]. This makes it harder for many older people to visit hospitals or health care facilities, and the need for eHealth services to provide health care at home has been increasing. The medical services offered by an internet-based platform have the advantage of increased equality in access to medical services during any type of crisis.

eHealth has been a World Health Organization priority since 2005. It defined eHealth as “a health-related field including medical and health services, health surveillance, health literature, health education, knowledge, and research” and has provided international reports on eHealth readiness [3]. In recent years, eHealth has been increasingly used as a generic term that covers a variety of mobile health (mHealth), telemedicine, and telehealth services, as well as eHealth data management [4]. eHealth is becoming an important solution for people who need to consistently manage their health even at home and receive immediate professional medical services by providing low-cost and high-quality health care [3].

The development of the Internet of Things (IoT) has contributed to advances in the eHealth field. IoT is a technology that allows physical objects, devices, and computers to interact using networks to collect and exchange data [5]. Ambient assisted living in health care facilities using IoT is designed to help older adults’ lives. This allows older adults with chronic diseases to measure blood pressure, glucose, electrocardiogram, and body temperature, which need to be monitored every day, and the data can be immediately shared with medical facilities [6]. Recently, advances in sensors and machine learning have made it possible to better perceive and understand the daily lives of older adults. This could lead to the development of eHealth technologies that monitor daily health conditions, share information with health care facilities, and respond to emergencies. The improvement of these technologies can relieve the social burden of aging and accelerate the transition to personalized digital health care that can meet the needs of individuals seeking independent living [7].

However, older people’s acceptance, adoption, and use of technology have lagged behind that of younger people. In addition, older people may have low eHealth literacy or low ability to access, evaluate, and use health information to make medical decisions [8]. Nonetheless, as their health concerns and health care needs are higher than for any other age group, some studies have confirmed that a growing number of people from this demographic segment are accepting the technology and are willing to use it in the future [9]. The first step toward applying eHealth technology in the future and bringing it into real life is to identify the factors that older adults, as users of eHealth, consider important to embrace the technology [10].

In the process of expanding the use of new technologies, many studies have been conducted to find how users accept specific technologies. The 2 representative models are the Technology Acceptance Model (TAM), which posits that both perceived usefulness and perceived ease of use affect the user’s attitude and behavioral intention [11], and the Unified Theory of Acceptance and Use of Technology (UTAUT), which was designed as a synthesis of 8 major technology acceptance models [12]. There are many studies on eHealth acceptance for older adults that extend, transform, or combine these 2 models to identify acceptance factors [13-16]. However, neither is it easy to find evidence for an appropriate acceptance factor model, according to the conditions of the research nor is it easy to construct an optimized acceptance model.

**Figure 1:**
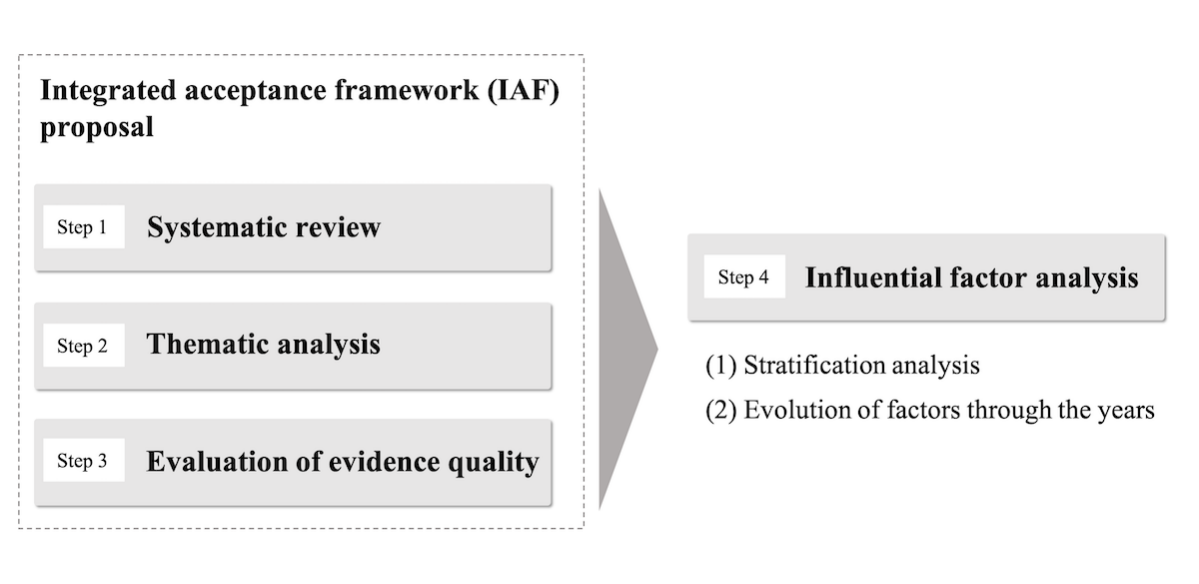
The 4-step study model.

For use in future studies in this field, this study extracts the acceptance factors from studies on eHealth for older people over the past 11 years through a systematic review. After that, we propose an integrated acceptance framework (IAF) that groups the extracted acceptance factors through thematic analysis. Then, the criteria for evaluating the evidence for each factor incorporated into the IAF are provided. Finally, the proposed IAF is analyzed according to the detailed conditions. This study proceeds in 4 steps, from data extraction for the IAF to analysis for the IAF application (Figure 1). One of the main aspects that differentiate IAF from TAM and UTAUT is the presentation of a wider range of factors and dimensions based on acceptance factors that have been covered in research over the past 11 years. Moreover, although TAM and UTAUT are generic acceptance frameworks, IAF is intended to be a framework specifically tailored for a concrete technology (eHealth) and a particular population (older people). In this way, it is expected to be a more useful tool for highlighting the potential barriers and facilitators when planning a new adoption scenario.

### Objective and Process

This study can contribute to eHealth research and industry in the following three ways: (1) the development of an IAF, which comprises acceptance factors grouped in dimensions for the analysis of eHealth acceptance in old age that emerge from the analysis of existing evidence; (2) a metric for the assessment of the quality of the evidence found in the systematic review study that allows for the normalization and integration of the evidence on the impact of acceptance factors across different and diverse studies; and (3) stratification analyses of the IAF application according to the participant’s health status and verification time and analysis of the evolution of the factors through the years.

## Methods

### Overview

We conducted a 4-step study (Table 1). First, the primary studies were selected through a systematic review, and the acceptance factors and basic data for analysis were extracted from the selected studies. Second, the extracted factors were grouped through thematic analysis, and a framework for acceptance was proposed. Third, the metrics for quality assurance were defined to evaluate the weights of the factors addressed in the study and apply them to the framework. Finally, we applied the resulting framework for different scenarios—first, according to the health, and second, according to the verification time—resulting in particularized IAFs. Moreover, we analyzed the changes in these factors over the years.

**Table 1:** Step-by-step study agenda.

Step and agenda				Description
**Systematic review**				
	Which research was selected through a systematic review, which research methods were used for each study, who were the participants, and for which technologies were the acceptance factors studied?		This allows a comprehensive review of research methods and research distribution of selected studies.	
**Thematic analysis**				
	What are acceptance factors verified through each study, and can the factors be grouped by thematic analysis to present an IAF^a^?		A quick overview of the selected studies indicates that similar elements are considered in different studies under different terms and with different levels of abstraction. The need to generate an IAF that would emerge from a thematic analysis of the collection of all acceptance elements mentioned in each study, thus grouping similar elements and providing a dimensional classification of acceptance factors, is anticipated.	
**Evaluation of evidence quality**				
	Can the importance of each acceptance factor be assessed by combining the evidence provided in different studies?		Given the high variability in the research methods used in the various studies and in the size and characteristics of the participants, the need to establish a metric that assesses the quality of the evidence provided by each study is anticipated. In addition, it is possible to compute a weighted combination of the importance of acceptance factors proposed in the selected studies.	
**Influential factor analysis**				
	**Stratification analysis for the IAF**			
		IAF by health status	Through this analysis, it is possible to compare the acceptance factors studied in the group of healthy older adults with the acceptance factors studied in the group of older adults with diseases.	
		IAF by verification time	The relevant acceptance factors of preadoption (before installation) and those of postadoption (after installation or after use) will be compared and analyzed.	
	Evolution of factors along the years		The analysis of whether there has been any change in acceptance factors over time, considering the rapidly developing eHealth technology and its growing adoption, is a goal of this research.	

^a^IAF: integrated acceptance framework.

### Step 1: Systematic Review

This study selected and analyzed the studies according to the PRISMA (Preferred Reporting Items for Systematic Reviews and Meta-Analyses) guidelines. The study scope was defined by the population, intervention, comparison, and outcomes (PICO) model, and accordingly, the search scope and research questions were defined. In the screening stage, 3 reviewers collaborated using the Covidence tool (Veritas Health Innovation Ltd). During the process of extracting and organizing data, a Microsoft Excel spreadsheet was used and shared for collaboration through Microsoft Teams.

The PICO model was applied to define the research scope and search strings as follows:

- Population: older adults who have the possibility of using eHealth
- Intervention: eHealth technology (including mHealth, telemedicine, and telehealth) that older users may experience or think about
- Comparison: comparison among the participants’ conditions, comparison based on verification time, and comparison of changes in acceptance factors by year
- Outcome: extraction and consolidation of acceptance factors and their impact on the adoption of eHealth services for older adults

Search strings were defined as combinations that can retrieve as many related studies as possible, with consideration given to PICO. Our final search string was *(ehealth OR telehealth OR mhealth OR uhealth OR health technology OR telemedicine) AND (older OR elderly OR senior) AND (adoption OR acceptance) AND (factors OR barriers OR determinants OR facilitators)*. The search scope was established as article title, abstract, and keywords. The databases used were Web of Science, Scopus, PubMed, IEEE, and MEDLINE. The review was conducted on conference papers or journal articles published during the 11 years from 2010 to 2020.

The set of studies collected through the search was finally selected using the following criteria and quality evaluation questions.

The exclusion criteria were as follows:

- Articles not written in English
- Articles that did not directly use the terms *acceptance* and *health technology* or related terms in the title, abstract, or entire text
- Studies that discuss eHealth adoption factors but not for older users
- Meta-analysis reviews the same subject

The quality evaluation questions were as follows:

- Are the influential factors clearly defined?
- Is the empirical evidence presented?
- Are the ages of the participants clearly stated (mean age of ≥60 years)?
- In the case of quantitative research, is the number of participant responses sufficient?
- In the case of qualitative research, has there been sufficient discussion of acceptance factors?

The data to be extracted from each study were defined according to the agendas in each step, as shown in Textbox 1. The data were extracted and organized in step 1, and the extracted data from each agenda were used in each step.

**Data definitions for extraction.**

**Year of publication**

- Year the study was published

**Country**

- Countries subjected to study

**Participants’ mean age**

- Average age of participants

**Verification time**

- When the acceptance factors are verified

**Study method**

- The methods used to study the acceptance factors

**Technology**

- Health-related technologies in studies

**Theory**

- Theories on which the study is based

**Participants’ condition**

- Participants’ health status or recruitment conditions

**Factor, barrier, or facilitator**

- Factors, barriers, and facilitators tested

**Result**

- Research results and insights

### Step 2: Thematic Analysis

The high number and diversity of factors, together with the variety of research methods, make it difficult to collect existing evidence and reach meaningful conclusions about the factors that really have an impact on the acceptance and adoption of these technologies. To overcome this, we formed an integrated framework for eHealth acceptance factors in older adults. All factors extracted from the selected studies by the systematic review process were defined as either positive or negative. Then, a thematic analysis process was conducted with the goal of identifying, analyzing, and interpreting patterns of meaning (or *themes*) within the set of original acceptance factors. We grouped them according to commonality in the meaning of the original acceptance factors in a bottom-up fashion. The 3 authors jointly analyzed and classified the acceptance factors through Microsoft Teams, and they reviewed and discussed each article to understand the meaning of the factors used in that article. Concretely, the first author conducted an initial thematic analysis after data extraction. Later, the other 2 authors participated in several consensus meetings. The other authors are senior researchers in two complementary disciplines: the first is a professor of computer science with a degree in psychology and extensive experience in acceptance models, and the second is a professor of biomedical engineering with wide experience in eHealth and older adults using technologies. After extracting and defining factors for 3 weeks, grouping was conducted according to themes for an additional 2 weeks.

### Step 3: Evaluation of Quality of Evidence

To assess the impact of the factors, we evaluated and reflected the quality of evidence in each study beyond the frequency of the factors used in the studies. For a systematic review, there is a grading of recommendations, assessment, development, and evaluation (GRADE) method that evaluates the quality of the evidence for each outcome by applying a set of evaluation criteria [17]. With GRADE, the quality of the evidence is evaluated according to the research method, as well as the risk of bias, inconsistency, indirectness, imprecision, and large magnitude of effect. However, GRADE focuses on the results of the study rather than evaluating the overall quality of the research and is mainly targeted for experiments related to health care. Consequently, we found it difficult to apply GRADE in our review. Thus, we only took the research methods criteria considered in GRADE and other studies [17,18], and we felt it was necessary to add some new criteria that could assess the quality of evidence of the selected studies. One of the outcomes of this research is a metric that defines a set of relevant evaluation criteria.

The impact of the acceptance factors was analyzed by calculating the evidence quality score of each study according to this metric and deriving from it the weight of the acceptance factors studied.

In this way, the proposed IAF was enhanced by reflecting the impact of acceptance factors.

### Step 4: Influential Factor Analysis of the IAF

We analyzed the IAF that resulted from the research to better understand the relative importance of the factors according to different conditions. To do this, a stratification analysis [19] was applied that allowed the classification and analysis of the factors according to the conditions. First, the factors that depend on the health condition of the participant were analyzed; then, the analysis was repeated according to the verification time (preadoption and postadoption). In addition, the IAF was analyzed by year to examine the evolution of factors over the years.

## Results

### IAF Steps

#### Step 1: Systematic Review

**Figure 2:**
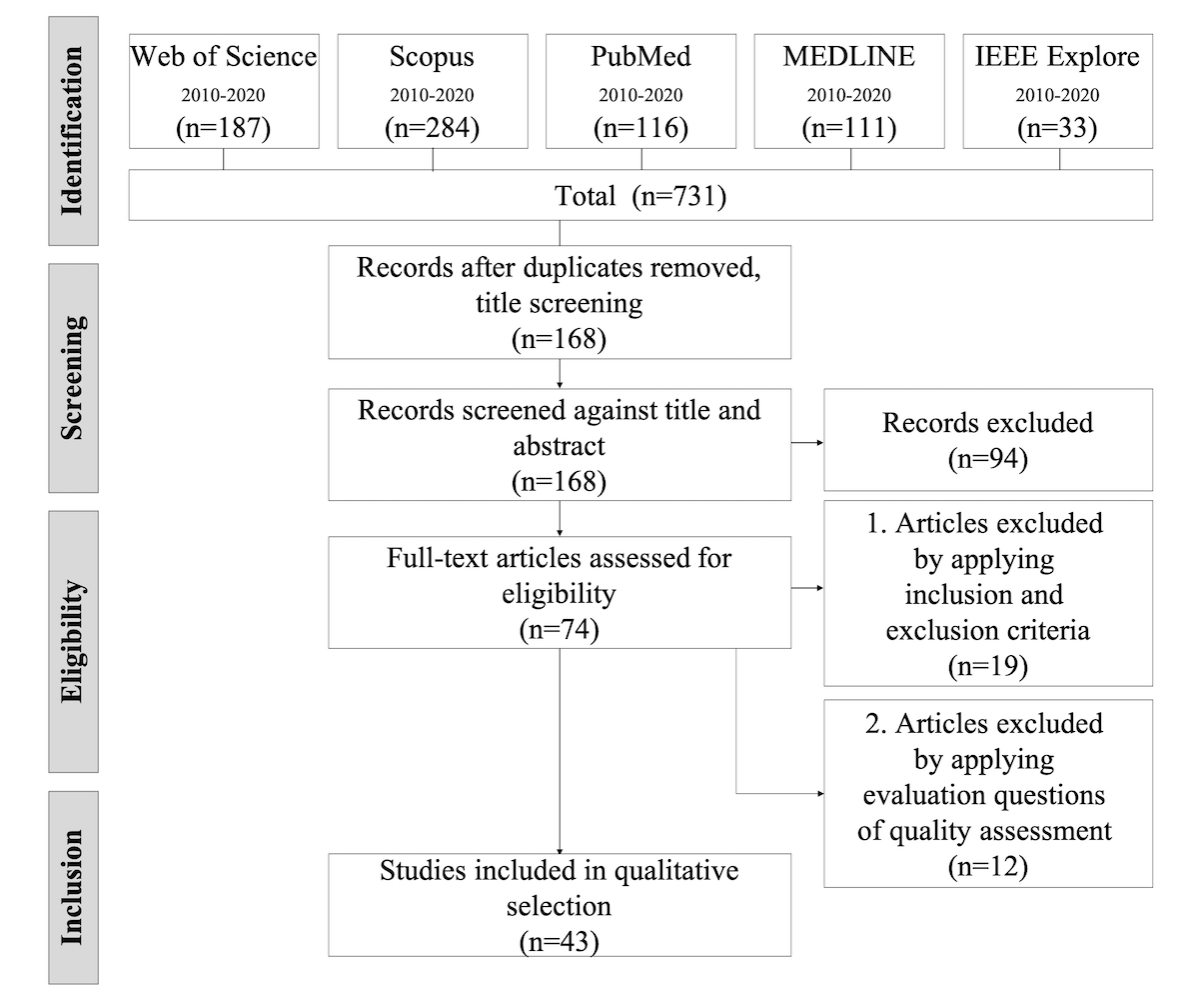
PRISMA (Preferred Reporting Item for Systematic Reviews and Meta-Analyses) flowchart for study selection.

According to the PRISMA guidelines, of the 731 studies retrieved from the databases, after excluding duplicates, 168 (23%) studies were screened. Of the 168 studies, after excluding 94 (55.9%) studies that were considered irrelevant, a total of 74 (44%) studies were reviewed for full text during the eligibility phase. Figure 2 shows the PRISMA flowchart of the study selection process, where 58% (43/74) of the articles were finally selected. The selected studies clearly identified acceptance factors for eHealth or health technology for the older population.

Multimedia Appendix 1 [13-16,20-58] lists the 43 studies and their corresponding basic data (see Textbox 1 for definitions). Of the 43 selected publications, 27 (63%) reported quantitative studies, 10 (23%) reported qualitative studies, and 6 (14%) reported mixed methods studies. Only 9% (4/43) of them were longitudinal studies, which observed and analyzed the same group for a long period, and their continuous observation period ranged from 3 months to 2 years. Quantitative research was mainly conducted as a survey in the form of mail or web-based questionnaires, and because of the characteristics of old age, there were also studies conducted through a face-to-face survey with explanations about the research. For qualitative research, in-depth interviews and focus group interviews were conducted at a similar rate.

Participants’ health conditions for each investigation were also identified. Of the 43 studies included in this research, 26 (60%) studies only considered participants without pre-existing disease conditions, followed by 9 (21%) studies that included participants with chronic diseases. Of the 43 studies, there was 1 (2%) study that compared healthy participants to participants with chronic diseases, and 2 (5%) studies compared healthy participants to participants with heart disease.

**Figure 3:**
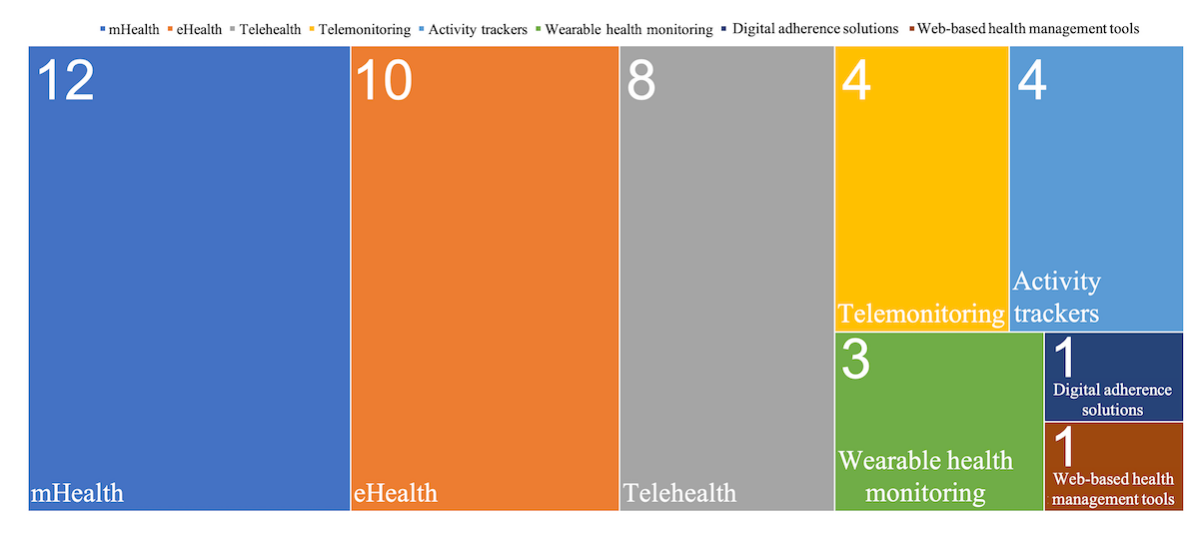
Target technology. mHealth: mobile health.

Figure 3 displays the distribution of health technologies that need to be studied. mHealth was the most common classification, with 28% (12/43) of studies, followed by eHealth with 23% (10/43) of studies, which dealt with general and integrative health technology.

Most studies were based on existing technology acceptance theories, 40% (17/43) of studies were based on TAM, and 21% (9/43) of studies were based on UTAUT.

The distributions of the selected 43 studies, according to country and year, are shown in Multimedia Appendix 2. Excluding an anomalous decrease in 2018, the trend is a growing number of papers, with recent studies in 2019 and 2020 accounting for a large proportion. The largest number of studies was conducted in the United States, followed by studies conducted in China.

#### Step 2: Thematic Analysis

**Figure 4:**
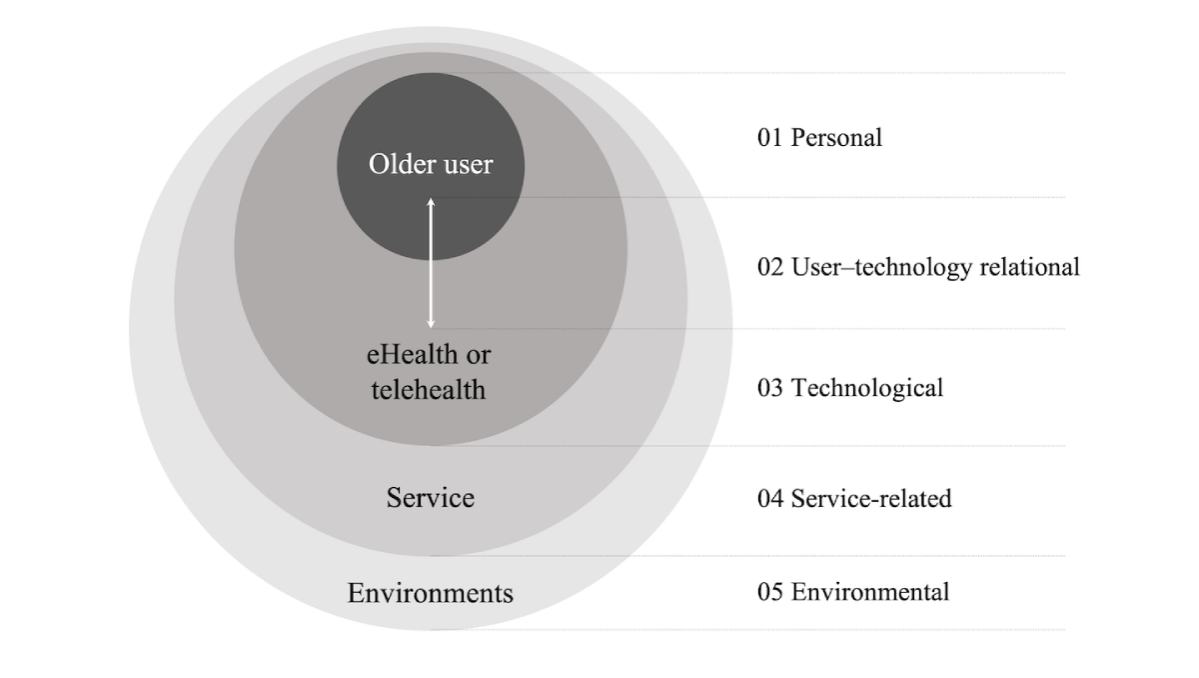
The 5 dimensions for acceptance factors.

A total of 105 acceptance factors were extracted from the studies. The 105×43 matrix relating acceptance factors to the studies was too sparse, and the absolute frequency of acceptance factors across the studies was too low. It was obvious that this could not be taken as the basis for the combination of evidence. Therefore, a thematic analysis process was conducted with the goal of identifying and analyzing the patterns of themes within the set of original acceptance factors. By grouping according to commonality in the meaning of original acceptance factors in a bottom-up fashion, the resulting IAF comprised 23 representative acceptance factors or themes, which were categorized into five dimensions: (1) personal, (2) user–technology relational, (3) technological, (4) service relational, and (5) environmental (Figure 4). The details of the 23 final acceptance factors, their corresponding elements (rephrasing the original acceptance factors), the frequency with which they are analyzed across the 43 studies, and the type of influence they have been found to exert on the decision to use eHealth (positive or negative), are included in Table 2.

The *personal* dimension comprises a total of five factors related to the user: (1) *personal characteristics*, which comprise an individual’s basic profile; (2) *personal condition* to reflect an individual’s health status or activity level; (3) *personal capabilities* to know eHealth acceptance capacities; (4) *personality and attitude*, which considers all personal traits, beliefs, and attitudes that can have an impact on the adoption of eHealth technology; and (5) *preferences*, which reflect personal inclinations for health care.

The *user–technology relational* dimension comprises five factors that lie in the intersection between the user and technology: (1) *how technology addresses user needs/characteristics* to consider the degree of matching between the technology and the real needs of the user; (2) *experience with technology* to take into account previous experience with other technologies; (3) *perceived usefulness of technology*; (4) *perceived ease of technology*; and (5) *attitude toward technology*, which groups concerns and feelings that the user has toward the technology.

The *technological* dimension comprises six factors related to the technology: (1) *features/functions* of technology, (2) *quality of technology and device*, (3) *usability* of technology, (4) *hedonic motivation* of technology, (5) *automaticity*, and (6) *benefits for users* of technology use.

The *service-related* dimension comprises a total of five factors that consider the service aspects in the adoption of eHealth technology: (1) *support for use*, (2) *cost for eHealth service*, (3) *service quality*, (4) *organizational factors* related to service operation, and (5) *alignment with government policies*.

Finally, the *environmental* dimension comprises two elements that address the context in which the user will adopt the technology: (1) *physical environments*, such as distance from hospitals and internet accessibility, and (2) *social influence*, which is influenced by people around older users.

The factors of *personality and attitude* in the *personal* dimension included most of the elements. These elements correspond to personal dispositions, personality traits, or attitudes that can influence the acceptance of new things or changes in the way they deal with health. The factor of *attitude toward technology* in the *user–technology relational* dimension comprises elements that mostly affect the acceptance of eHealth technology in a negative way, such as technology anxiety, privacy concerns, and security concerns.

The *personal characteristics* factor, the element of chronic health condition or health status in the *personal condition* factor, and the element of the degree of satisfaction with existing medical services in the *how technology addresses user needs/characteristics* factor have shown both positive and negative effects on eHealth acceptance, depending on the study.

Thus, we propose an IAF that comprises these 23 grouped factors and 5 dimensions to be used as a reference in future research on eHealth acceptance.

**Table 2:** Proposed integrated acceptance framework with 23 acceptance factors and their elements (N=43).

Factor and element				Frequency, n (%)		Influence		
						Positive		Negative
**Personal**								
	**Personal characteristics**							
		Age	6 (14)		✓		✓	
		Gender	5 (12)		✓		✓	
		Educational background	4 (9)		✓		✓	
		Lifestyle and residence type	4 (9)		✓		✓	
		Income	2 (5)		✓		✓	
		Work status	2 (5)		✓		✓	
		Adequate financial status	1 (2)		✓			
		Geographical location	1 (2)		✓		✓	
		Health knowledge	1 (2)		✓			
	**Personal condition**							
		Chronic health condition or health status	8 (19)		✓		✓	
		High activity level	1 (2)		✓			
		Independence	1 (2)		✓			
	**Personal capabilities**							
		Self-efficacy or competence	9 (21)		✓			
		Decreased physiological or cognitive capability	6 (14)				✓	
		Participation	1 (2)		✓			
	**Personality and attitude**							
		Concerns about risk	5 (12)				✓	
		Conversion readiness or personal innovativeness	4 (9)		✓			
		Resistance to change	2 (5)				✓	
		Personal proactivity	2 (5)		✓			
		Sense of control	2 (5)		✓			
		Confidence in control of health	1 (2)		✓			
		Overanxiety about health	1 (2)				✓	
		Perceived social risk	1 (2)				✓	
		Need for cognitive closure	1 (2)				✓	
		Willingness to take a chance	1 (2)		✓			
		Ability to take advantage of opportunities	1 (2)		✓			
		Self-esteem	1 (2)		✓			
		Self-confidence	1 (2)		✓			
		Reluctance to rely on a machine	1 (2)				✓	
	**Preferences**							
		Preference for face-to-face contact	3 (7)				✓	
**User–technology relational **								
	**How technology addresses user needs or characteristics**							
		Lack of needs	4 (9)				✓	
		Degree of satisfaction with existing medical service	2 (5)		✓		✓	
		Insufficient contents or functions	1 (2)				✓	
		Needs are already addressed by caregiver	1 (2)				✓	
		Desire for ownership of and access to medical information	1 (2)		✓			
		Information or system feature overload	1 (2)				✓	
		Health care needs	1 (2)		✓			
	**Experience with technology (literacy)**							
		Lack of information and awareness	7 (16)				✓	
		Prior experience with technology	6 (14)		✓			
		eHealth literacy	4 (9)		✓			
		Poor eHealth experience	3 (7)				✓	
		Frequency of internet use	1 (2)		✓			
	**Perceived usefulness of technology**							
		Perceived usefulness	20 (47)		✓			
		Performance expectation	8 (19)		✓			
		Perceived security	7 (16)		✓			
		Perceived compatibility	4 (9)		✓			
		Perceived ubiquity	1 (2)		✓			
		Perceived relative advantage	1 (2)		✓			
	**Perceived ease of technology**							
		Perceived ease of use	18 (42)		✓			
		Difficulty with new technology	8 (19)				✓	
		Effort expectation	8 (19)		✓			
		Perceived complexity of technology	2 (5)				✓	
		Amount of perceived effort	1 (2)				✓	
	**Attitude toward technology**							
		Technology anxiety	13 (30)				✓	
		Privacy concerns	8 (19)				✓	
		Lack of interest	4 (9)				✓	
		Security concerns	4 (9)				✓	
		Lack of trust in service	4 (9)				✓	
		Trust in service	3 (7)		✓			
		Negative feeling about constant monitoring	1 (2)				✓	
**Technological**								
	**Features or functions**							
		Track vital signs or monitor my information	3 (7)		✓			
		Functions to help existing health care services	2 (5)		✓			
		Monitor health trends	1 (2)		✓			
	**Quality of technology and device**							
		Technology instability	4 (9)				✓	
		Convenience	2 (5)		✓			
		Physical comfort (wearable)	1 (2)		✓			
	**Usability**							
		Insufficient user-friendliness	6 (14)				✓	
		Learning difficulty of new technology	2 (5)				✓	
		Lack of instructions	2 (5)				✓	
		Esthetics	1 (2)		✓			
		Helpful instructions	1 (2)		✓			
	**Hedonic motivation**							
		Hedonistic motivation	1 (2)		✓			
	**Automaticity**							
		Using it everyday	1 (2)		✓			
		Using a variety of functions	1 (2)		✓			
		Habit	1 (2)		✓			
	**Benefits for user**							
		Share data with someone	3 (7)		✓			
		Digital solutions that remove personal barriers	2 (5)		✓			
		Medical records in one place	2 (5)		✓			
		Observation of changes after use	2 (5)		✓			
		Portable personal records	1 (2)		✓			
		Prevention of unnecessary tests or medical accidents	1 (2)		✓			
**Service related**								
	**Support for use**							
		Technical support	6 (14)		✓			
		Support from people around me	6 (14)		✓			
		Peer support	6 (14)		✓			
		Adequate training	4 (9)		✓			
		Intergenerational support	4 (9)		✓			
		Support from service	4 (9)		✓			
		Hospital support	2 (5)		✓			
		Not enough support for technology use	1 (2)				✓	
	**Cost for eHealth service**							
		Cost burden	9 (21)				✓	
		Service affordability	4 (9)		✓			
		Service availability	3 (7)		✓			
		Price value	2 (5)		✓			
	**Service quality**							
		Information quality or service quality	2 (5)		✓			
	**Organizational factors**							
		Care assistance center linked to service	1 (2)		✓			
		Improvement of health care interactions	1 (2)		✓			
		Provided in parallel with existing direct visits	1 (2)		✓			
	**Alignment with government policies**							
		Government policy	1 (2)		✓			
**Environmental**								
	**Physical environments**							
		Internet connection instability	3 (7)				✓	
		Distance to hospital	1 (2)		✓			
	**Social influence**							
		Social norms or subjective norm	12 (28)		✓			
		Physician’s recommendation	6 (14)		✓			
		Recommendation from people around me	5 (12)		✓			
		Family recommendation	1 (2)		✓			
		Close people’s eHealth readiness	1 (2)		✓			

#### Step 3: Evaluation of Quality of Evidence

##### Overview

The assessment results should quantify the reliability of the findings on acceptance factors discussed in each study and whether their conclusions can be confidently applied to future related studies. The proposed metric is based on three criteria:

1. Reliable methodology
2. Participant’s experience with the specific target technology
3. Research and publication year

The score for each of the 3 criteria ranged from 1 to 4: very low=1, low=2, moderate=3, and high=4. As studies with *very low (1 point)* quality by these criteria have already been excluded through the quality assessment of the full text, the selected studies received scores ranging from 2 to 4 points.

##### Reliable Methodology

The quality of evidence increases when the applied research methods can provide high internal and external validity. It is considered that the validity of quantitative studies strongly depends on the number of participants. We also considered that mixed approaches, in which quantitative and qualitative methods are combined, tend to have higher validity than single methods. Finally, the clarity and reliability of the analysis method also have an influence on validity. The rules applied for the assessment were as follows:

- High (4 points): studies with multidimensional approaches that applied a mixed or longitudinal study
- Moderate (3 points): quantitative studies with sufficient participants and clear analysis methods; qualitative studies following a reliable analysis method
- Low (2 points): quantitative research with <100 participants; qualitative research that did not mention a clear analysis method

##### Participant’s Experience of the Specific Target Technology

This criterion evaluates whether the target technology was clearly explained to or experienced by the participants before discussion and investigation. With a high degree of understanding of the technology being studied, participants could express their intentions more accurately. Otherwise, their answers could be biased by misunderstandings or prejudices. The rules applied for the assessment were as follows:

- High (4 points): The subject of investigation clearly recognized the target technology through a prototype or demonstration video, or the target technology was used for a certain period.
- Moderate (3 points): The technology was presented through text, images, or explanations from the investigator. Alternately, participants had an indirect understanding of the target technology based on their previous experiences with other technologies.
- Low (2 points): The method for the presentation of the target technology to the participants was not mentioned in the article.

##### Publication Year

Recently, eHealth has been developing at a faster rate. In this context, additional points were applied to recent research in consideration of the fact that acceptance factors can be affected by recent advancements in technology and infrastructure. Moreover, the adoption of eHealth technologies has been increasing over time, possibly leading to a change in the influence exerted by some acceptance factors.

- High (4 points): studies from 2017 to 2020
- Moderate (3 points): studies from 2014 to 2016
- Low (2 points): studies from 2010 to 2013

##### Quality of Evidence

We followed four steps to calculate the influence of the acceptance factors:

1. Each study obtained a quality score according to the defined metric, with the results ranging from a maximum of 12 points to a minimum of 8 points.
2. This score was normalized by transforming a perfect score of 12 into 1 and a score of 8 into 0.67.
3. The occurrence of each factor in a study was represented by the normalized score for the corresponding study.
4. The values for all occurrences of a factor were totaled.

**Figure 5:**
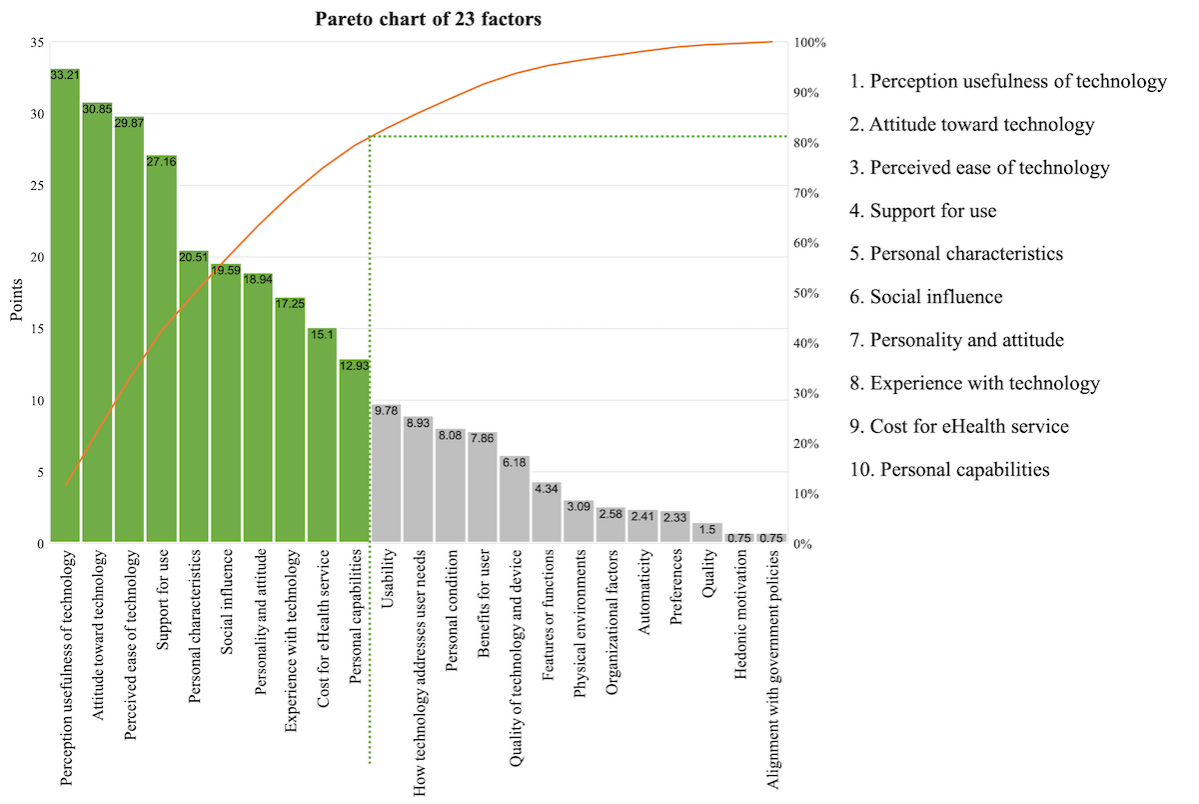
Factor impact based on quality assessment of evidence on integrated acceptance framework.

Through this process, the real influence of the acceptance factors was measured in a more reliable way than just by considering the absolute frequency. Figure 5 shows the impact order of the factors, reflecting the quality of the supporting evidence. The Pareto chart allows for the selection of important elements in the 80% criterion by cumulative impact [59]. Applying the Pareto chart, 10 factors can be distinguished among the 23 factors in the IAF, as shown in the chart (Figure 5): *perceived usefulness of technology*, *attitude toward technology*, *perceived ease of technology*, *support for use, personal characteristics, social influence, personality and attitude, experience with technology, cost of eHealth service, and personal capabilities*. Together, these account for 80% of the found evidence of impact.

**Figure 6:**
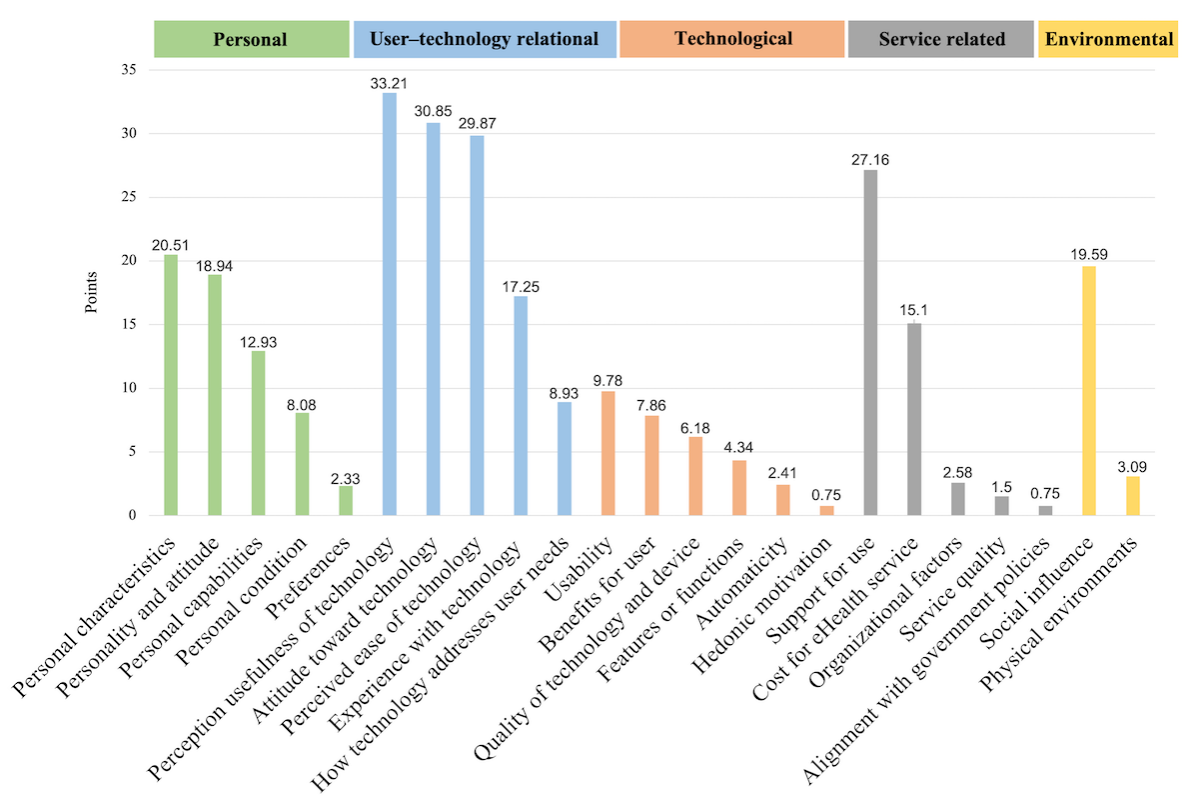
Factor impact on each dimension of integrated acceptance framework.

Figure 6 depicts the impact of the factors classified according to the 5 dimensions of the IAF.

The weights of the 5 dimensions were calculated as the sum of the weights of the factors corresponding to each dimension. As shown in Figure 6, as the weights of the factors constituting each dimension are different, the weights of each dimension are also different.

The acceptance factors in the *user–technology relational* dimension were considered the most important, and *support for use* in the *service-related* dimension, *personal characteristics* in the *personal* dimension, and *social influence* in the *environmental* dimension were also determined to be important. The *technological* dimension was evaluated to be less important than the other dimensions.

These impact scores for the factors in the IAF come to complete the framework and define the relative importance of each factor.

### Influential Factor Analysis

#### Overview

This section presents the results of the fourth step. The IAF proposed through the previous 3 steps was analyzed under 3 conditions. The following subsections describe the optimized IAF for each specific situation.

#### Stratification Analysis of IAF by Health Status

**Figure 7:**
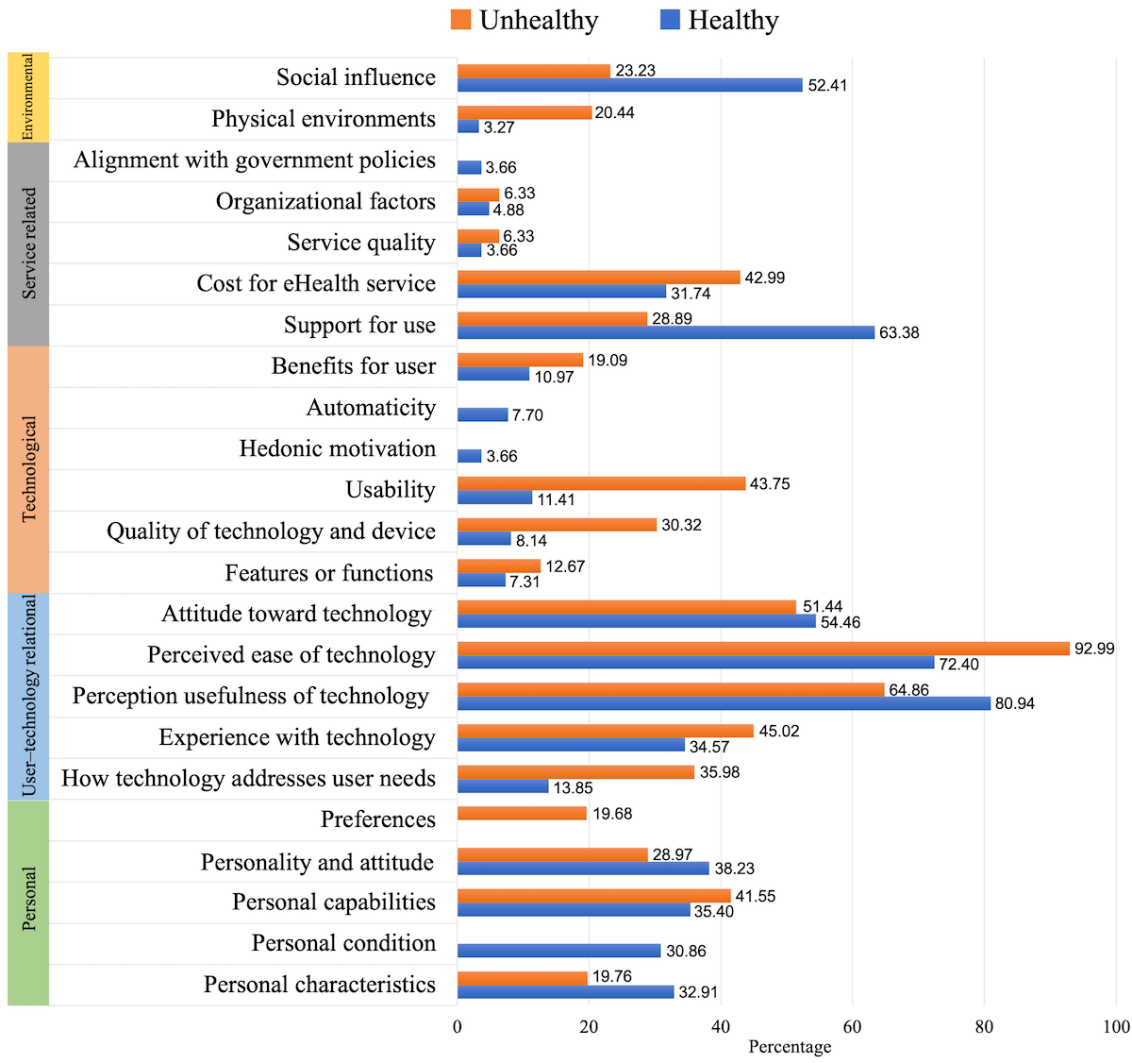
Comparison of the acceptance factor impact by participant health status.

As previously stated in step 1, of the 43 studies, 14 (33%) involved older adults with specific diseases (eg, chronic diseases and heart disease), and 26 (60%) included older adults without disease conditions. In addition, 7% (3/43) of studies [46,47,55] compared the healthy group and the group with diseases. It may be a risk to conclude that the participants without a disease condition are healthy; however, it is possible to determine that they are healthy compared with the group with a specific disease. Therefore, for the comparison based on participants’ conditions, the studies with older people without disease conditions were classified as the healthy group, whereas studies considering older people with diseases were classified as the unhealthy group, and the differences in the acceptance factors of these 2 groups were analyzed (Figure 7).

After applying the quality score of each study’s evidence, the impact of factors was calculated as a percentage, as the number of studies in each group was different. As a result, the most important factor in studies in the healthy group is the *perceived usefulness of technology*. In addition, the following factors were considered more important for the healthy group than for the unhealthy group: *support for use*, *social influence*, *perception of technology*, *personality and attitude*, and *personal characteristics*. However, the *preferences* factor in the IAF was not considered at all in the studies for this group. On the other hand, in the case of the unhealthy group, the most important acceptance factor was the *perceived ease of technology*. The factors *physical environment*, *cost of eHealth service*, *usability*, *quality of technology and device*, *perceived ease of technology*, and *personal capabilities* were considered more important for this group than for the healthy group. In this group, *personal condition*, *hedonic motivation*, *automaticity*, and *alignment with government policies* were not considered as acceptance factors. It was confirmed that the 2 groups showed different eHealth acceptance factor patterns. These results can be interpreted as evidence of variance in acceptance factors according to participants’ health conditions.

**Figure 8:**
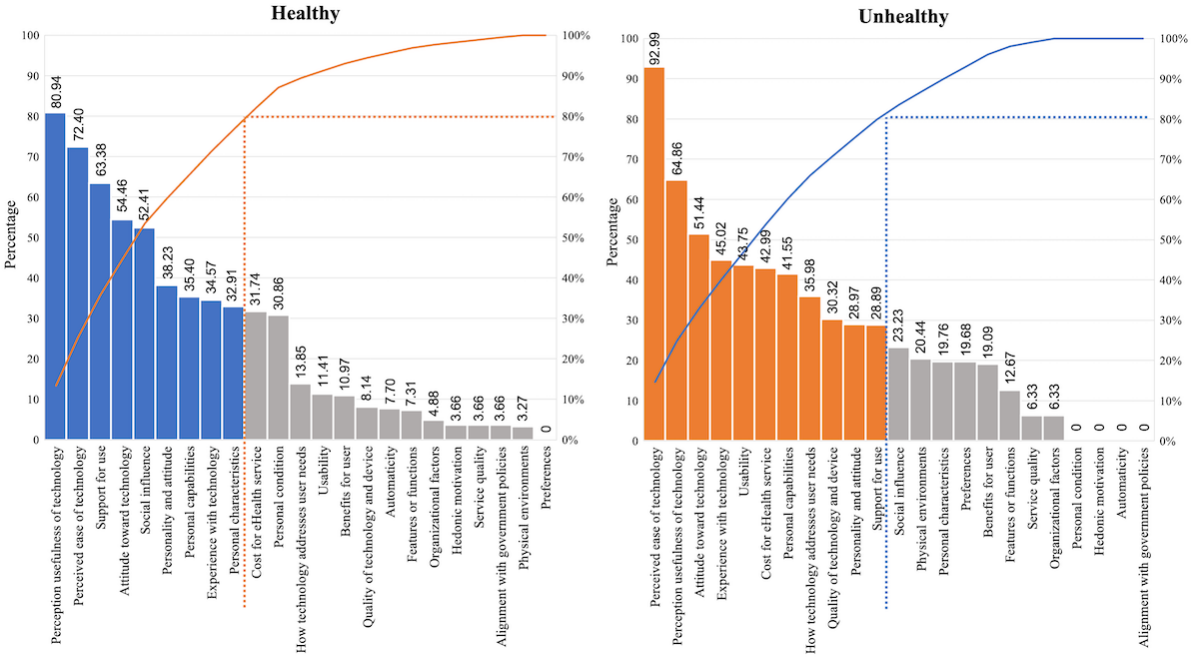
Pareto chart for integrated acceptance framework by participant health status.

Through the Pareto chart (Figure 8), nine acceptance factors for the healthy group IAF were identified: *perceived usefulness of technology, perceived ease of technology, support for use, attitude toward technology, social influence, personality and attitude, personal capabilities, experience with technology*, and *personal characteristics*. In addition, the following factors for studies on participants with diseases were found: *perceived ease of technology, perceived usefulness of technology, attitude toward technology, experience with technology, usability, cost of eHealth service, personal capabilities, how technology addresses user needs, quality of technology and device, personality and attitude, and support for use*.

#### Stratification Analysis of IAF by Verification Time

**Figure 9:**
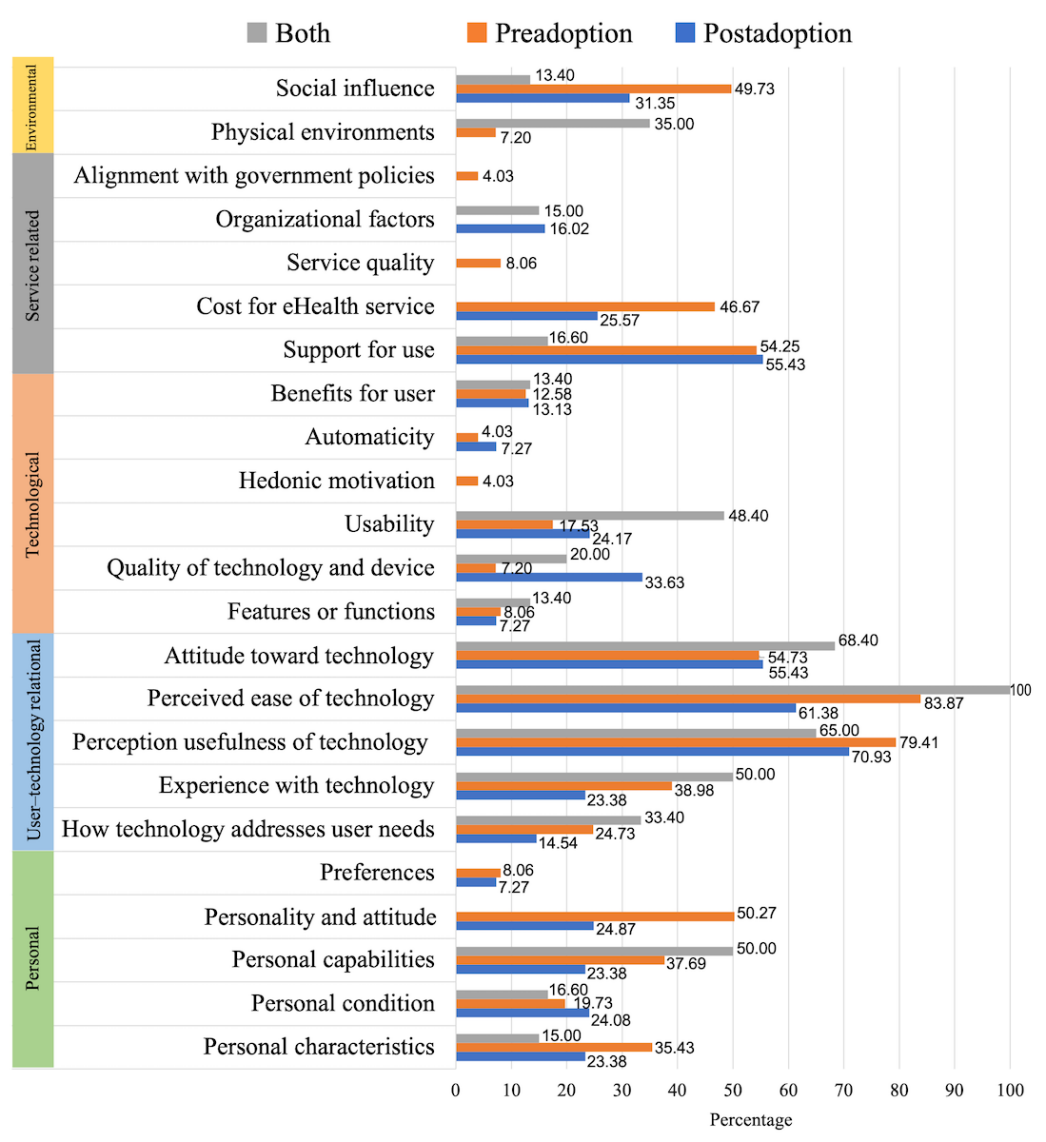
Comparison of the impact of acceptance factors by verification time.

We also analyzed the acceptance factors according to the time at which they were verified. In the results for step 1 (Multimedia Appendix 1), the verification time of the studies was divided into three stages: (1) before installation, (2) after installation or at the beginning of use, and (3) after enough use. In this analysis, we decided to compare preadoption and postadoption so that *before installation* was classified as preadoption, and the rest (*after installation/at the beginning of use* and *after enough use*) were classified as postadoption. The 43 studies were divided into 24 (60%) studies related to preadoption, 13 (30%) studies related to postadoption, and 6 (14%) studies that dealt with both situations. The acceptance factors according to the verification time were compared and analyzed, as shown in Figure 9.

The most important factor in the preadoption stage is the *perceived ease of technology*. The factors of *social influence, cost of eHealth service, perception of technology usefulness, personality and attitude*, and *personal characteristics* are more important at this verification time in studies on preadoption than in studies on postadoption. Moreover, the *organizational factors* from the IAF were not considered at all in the preadoption studies. The key factor for postadoption is the *perceived usefulness of technology*. *Organizational factors, support for use, quality of technology and device*, and *personal conditions* are more important acceptance factors in studies on postadoption than in studies on preadoption. At this verification time in the selected studies, the factors of *service quality*, *alignment with government policies*, and *physical environments* were not addressed as acceptance factors. As such, it is confirmed that the degree of influence of the acceptance factors from the IAF differed depending on the verification time.

**Figure 10:**
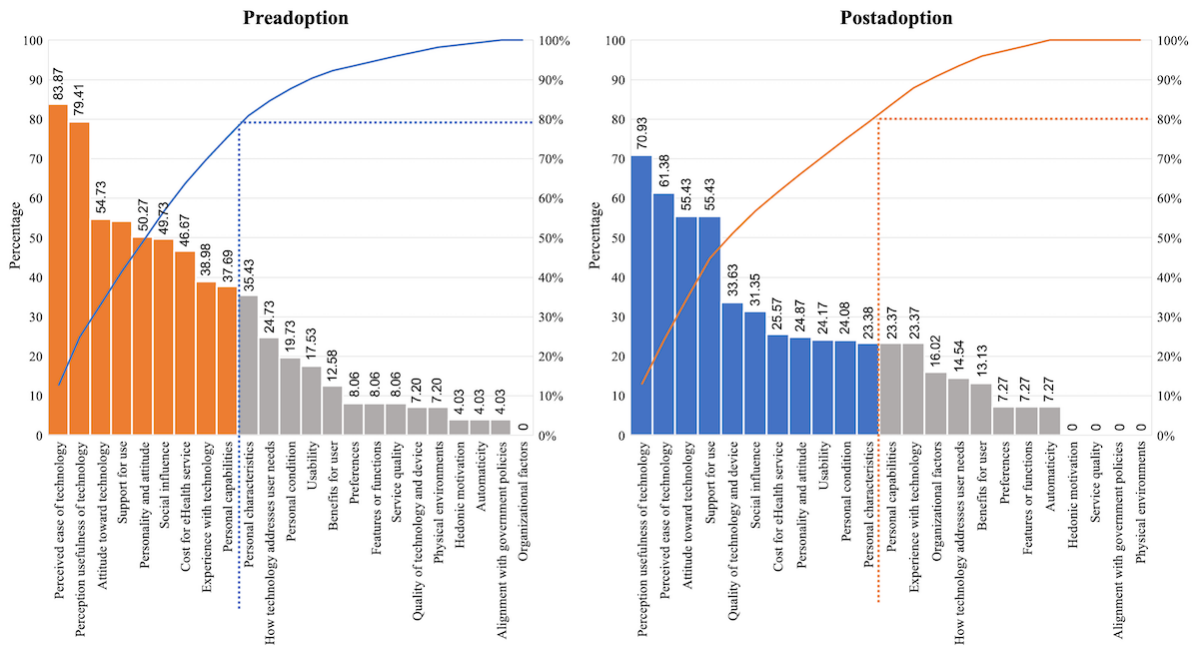
Pareto chart for integrated acceptance framework by verification time.

Applying Pareto (Figure 10), nine acceptance factors for the preadoption phase were identified: *perceived ease of technology, perceived usefulness of technology, attitude toward technology, support for use, personality and attitude, social influence, cost of eHealth service, experience with technology*, and *personal capabilities*. The top 11 factors for the postadoption phase were also identified: *perceived usefulness of technology, perceived ease of technology, attitude toward technology, support for use, quality of technology and device, social influence, cost of eHealth service, personality and attitude, usability, personal conditions, and personal characteristics.*

#### Analysis on Evolution of Factors Along the Years

**Figure 11:**
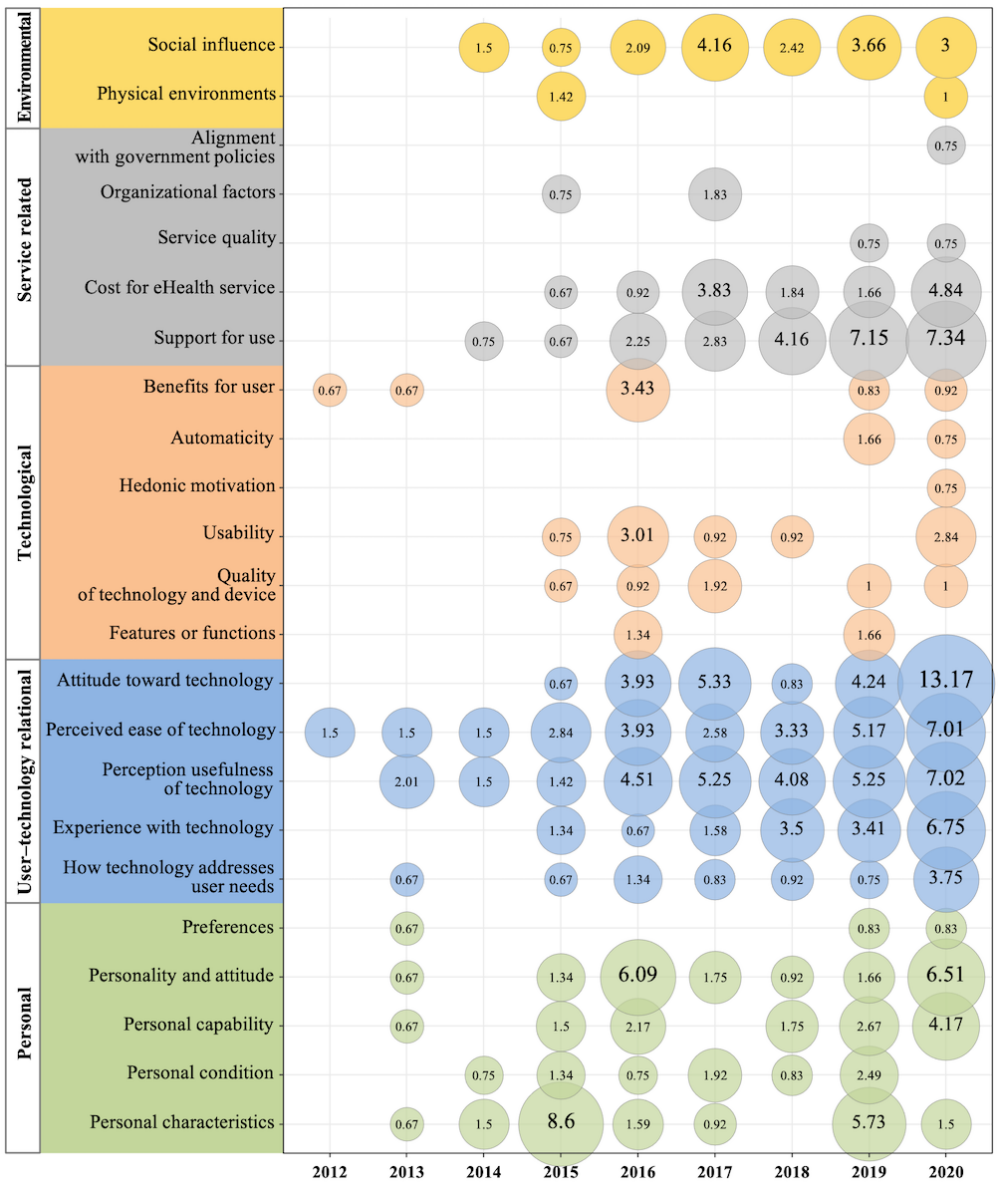
Impact change on integrated acceptance framework by year.

As shown in Figure 11, the acceptance factors that become more important with the passage of time are identified by analyzing the changes in the influence of the IAF factors per year. In the *personal* dimension, the pattern change was not very remarkable; however, it shows that the consideration for the *personal capability* factor has recently increased.

The *user–technology relational* dimension is more consistently considered than the other dimensions. In particular, the *perceived ease of technology* and the *perceived usefulness of technology*, which are addressed by the TAM and UTAUT models, are factors that have been steadily considered since the beginning of 2010. In addition, in the *user–technology relational* dimension, the factors of *experience with technology* and *attitude toward technology* have been mentioned since late 2015, and these factors have been considered more recently as well. Although it was difficult to find clear change patterns in the *technological* and *environmental* dimensions, the *support for use* factor in the *service-related* dimension has been identified as a key factor in recent years.

**Figure 12:**
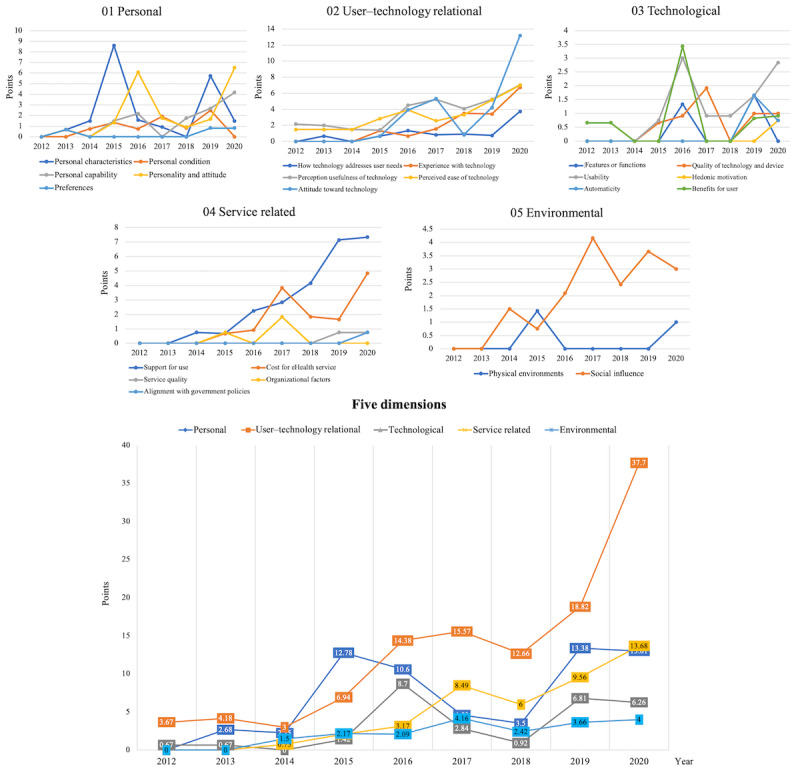
Linear change in the influence of acceptance factors by year.

Figure 12 illustrates the changes in the factors within each dimension through a linear graph, along with the accumulated changes in the 5 dimensions per year. The *user–technology relational* dimension has gained importance recently, and the importance of the *service-related* dimension has been steadily rising.

## Discussion

### Definition of an IAF

In step 1, we conducted a systematic review to select qualified studies and extract the data as the basis for the IAF. The basic data from the research were extricated, and those became the base material for the formation of the various comparative groups and the assessment of the evidence quality for the selected studies. A previous integrative review on the adoption of telehealth in older age [60] extracted facilitators and barriers through the Whittemore and Knafl [61] 5-stage methodology. However, to be applied to future research, a more comprehensive perspective and a detailed guide were needed. Thus, our study not only conducted data extraction and analysis from the selected studies but also presented an integrated framework, the IAF, that can be applied to future works.

In step 2, the factors and dimensions were defined. Existing studies have proposed various frames for analyzing the acceptance factors of technologies. For instance, Schulz et al [62] presented three aspects for understanding aging and technology as follows: (1) user characteristics, (2) technology functions, and (3) social factors. Our study presents five dimensions to analyze the acceptance factors for eHealth: *personal, user–technology relational, technological, service-related*, and *environmental*. Compared with existing research, our framework considers not only the main dimensions (user, technology, and environment) but also the issues that emerge as a result of the interaction between the main dimensions. As can be seen in the results of the analysis (step 3; Figure 6), the acceptance factors in the *user–technology relational* dimension were most significantly addressed by the reference studies, and this dimension is estimated to be a category that should be treated as important in future studies (step 4; Figures 11 and 12). Similarly, the *service-related* dimension reflects the fact that eHealth technologies are not isolated but integrated into health services that will also be transformed as a result of the adoption of the technology. This dimension makes visible the relevant interaction between the technology and the environment and the impact of this interaction on the user. The proposed 23 acceptance factors and 5 dimensions emerged from the existing evidence by thematic analysis in step 2, forming a framework. This IAF contributes to the state of the art a finer-grained analysis tool that integrates the diversity of elements considered in previous research and provides a common vocabulary.

In step 3, a new metric for evaluating the quality of the existing evidence was presented to expand the IAF with information about the impact of the different factors. Various methods for evaluating the quality of evidence in systematic reviews for health care have been studied [17,18]. A high score obtained through this evaluation method means that there are many confirmations that the actual effect is similar to the effect estimated by the study, whereas a low score means that the actual effect may be significantly different from the estimated effect [17]. In the systematic review on health information technology adoption [18], a ranking of 5 steps was applied to evaluate the quality of evidence, considering the study design and research method.

Although the research method and design are of paramount importance for judging the quality of evidence, additional criteria were needed that were more aligned with our analysis objective. Thus, three primary criteria were suggested by this study: the reliability of the research method, the degree of understanding of the target technology, and the year of the study. These 3 indicators contain detailed evaluation criteria that can be applied to other systematic studies as well. It is a reproducible and transparent framework for evaluating the certainty of the evidence, which minimizes author bias. Through this evaluation, the impact of acceptance factors was organized and analyzed. We believe that this is a more meaningful analysis than the assessment of the impact of the acceptance factors by just the frequency with which they were considered in previous studies. Once the impact of the acceptance factors has been incorporated into the IAF, it becomes evident that not all 23 factors are equally important for the acceptance of eHealth technologies by older users. A classical Pareto analysis was performed to select the set of highest priority factors (those that together account for 80% of the total impact). This is valuable information when facing a concrete situation seeking the adoption of a specific eHealth technology. This would help decision-makers to focus on available resources on the most influential factors.

### Adaptation of the IAF

In step 4, the diversity of adoption situations considered in the previous research was addressed. The goal was to propose a way in which IAF can be adapted to reflect existing evidence in specific adoption situations and investigate the extent to which IAF is a robust analysis framework. The differences in the IAF according to the health status of participants and the verification time at which acceptance factors were identified (before or after adoption) were compared through stratification analysis. We concluded that the highest priority acceptance factors vary according to the target’s health status. In addition, the data from this analysis illustrate the key acceptance factors in the IAF according to the health status of the users. Further diversity in user profiles could not be analyzed in the existing evidence. It would be interesting to explore in future research the way in which the weights of factors and dimensions differ for the same product or service based on the profile of the users.

The differences in the IAF according to the verification time were examined as well. In a previous study on the acceptance of electronic technology for older users [63], the acceptance factors of the preimplementation and postimplementation stages were compared and analyzed. Some of the factors were considered regardless of the stage; however, the acceptance factors that differed for each stage were also identified. In the same manner, in this study, we confirmed that there is a difference between the acceptance factors found relevant in the preadoption and postadoption phases of eHealth. This result confirms that it is necessary to apply the differentiated acceptance factors to different verification times.

We were also concerned about the validity of aggregating evidence about acceptance factors coming from studies performed in different years. Recently, eHealth technology has been further developed, and investment per country has increased significantly [3]. In addition, as the use rates of smartphones and the internet in older adults were different 10 years ago than they are in the present, we assumed that there would be a difference in acceptance factors with the passage of each year. As a result of comparing the acceptance factors by year in our last analysis, the continuous increase of the acceptance factors in the *user–technology relational* and the *service*-*related* dimensions was confirmed, except in 2018, a year in which there were few selected studies. These results support the inclusion of both dimensions in the IAF. In particular, the factors of *experience with technology* and *attitude toward technology* in the *user–technology relational* dimension have recently been dealt with as important, and the *support for use* factor in the *service-related* dimension is also a factor that has become more important in recent years. This result implies that one of the things a service provider needs to care about is ensuring that appropriate technical training is provided so that older users can use this equipment as efficiently as possible [62]. Although there is a belief that older people are not interested in using technology, many studies reveal that the facts prove otherwise and, more importantly, that there is a barrier to use because of a lack of adequate training and technical support [9]. When support, human factors, and stable technology are well combined, the barriers can be overcome. The results of this analysis identify the acceptance factors that have been becoming more important and suggest trends that can guide to set the direction for future studies.

### Limitations

To ensure the validity of this study, three threats were considered: (1) selection bias in systematic reviews, (2) threats to the extraction of acceptance factors, and (3) limitations in assessing the evidence quality.

#### Selection Bias

There is a risk that individual bias will be reflected in the research selection. To minimize this, the 4 exclusion criteria and 5 evaluation questions were defined. On the basis of these criteria, significant effort was put into the process of selecting high-quality studies that are suitable for the subject, following the PRISMA process. During the study selection process, Covidence was used to thoroughly verify that the selection was not based on the individual opinions of the 3 authors.

#### Threats to the Extraction of Acceptance Factors

There is a potential threat that the terms used to explain the acceptance factors may have been used to convey different meanings in different studies. For the initial 105 factors extracted, the authors made an effort to identify the exact meaning of the terms used within the context of each study. The factors that could cause semantic confusion were analyzed through a second review and discussion of the original studies by the authors. In addition, the conjunction of expertise of the authors in different disciplines minimized a possible bias related to a limited view of the technology and themes extracted.

#### Limitations in Assessing the Quality of Evidence

There is a potential risk of bias in the assessment of the quality of evidence in each study. As a form of prevention, the evaluation criteria used in the previous studies were thoroughly analyzed, and the evaluation criteria proposed for this review were established to be as specific as possible. In addition, to increase the confidence that the same results can be obtained even after re-evaluation, there was a focus on objectifying the criteria.

### Conclusions

Plans for and investments in eHealth are expanding worldwide. This is considered a good solution for covering places where traditional health care services do not reach [3]. The application and development of eHealth are growing more important because of the continuous increase in the older population and the availability of solutions. The continuous monitoring of older adults can reduce sudden accidents and help respond immediately to emergencies [64]. Despite their needs, the use of eHealth technology is a new challenge for older people. To pervade in daily life, not only must the technical, service-related, and environmental infrastructures of the eHealth service be prepared but also the personal factors and user–technology relational factors be considered. This is the key to understanding and addressing the needs and characteristics of users more clearly. This study systematically reviewed the research that evaluated the acceptance factors for older people in the eHealth service field over the last 11 years. In addition, this study proposed an IAF through thematic analysis and the assessment of the impact of these factors. In addition, the eHealth acceptance factors were compared and analyzed according to the participants’ health conditions, verification time, and year. We expect that the IAF will become a tool that can be used to predict the main barriers to be overcome and facilitators to be leveraged. These data will form a good research material base for the application of eHealth to older users in the future.

## Appendix

Multimedia Appendix 1 Study overview and methods.

Multimedia Appendix 2 General distribution of the studies.

## Abbreviations

GRADE: grading of recommendations, assessment, development, and evaluation
IAF: integrated acceptance framework
IoT: Internet of Things
mHealth: mobile health
PICO: population, intervention, comparison, and outcomes
POSITIVE: Maintaining and Improving the Intrinsic Capacity Involving Primary Care and Caregivers
PRISMA: Preferred Reporting Items for Systematic Reviews and Meta-Analyses
TAM: Technology Acceptance Model
UTAUT: Unified Theory of Acceptance and Use of Technology

## References

[1] (2018). Ageing and health. World Health Organization.

[2] (2021). COVID-19 risks and vaccine information for older adults. Centers for Disease Control and Prevention.

[3] (2016). Global diffusion of eHealth: making universal health coverage achievable: report of the third global survey on eHealth. World Health Organization.

[4] Eysenbach G (2001). What is e-health?. J Med Internet Res.

[5] Farahani B, Firouzi F, Chang V, Badaroglu M, Constant N, Mankodiya K (2018). Towards fog-driven IoT eHealth: promises and challenges of IoT in medicine and healthcare. Future Gener Comput Syst.

[6] Nazir S, Ali Y, Ullah N, García-Magariño I (2019). Internet of things for healthcare using effects of mobile computing: a systematic literature review. Wirel Commun Mob Comput.

[7] Kańtoch E, Kańtoch A (2020). What features and functions are desired in telemedical services targeted at polish older adults delivered by wearable medical devices?-Pre-COVID-19 flashback. Sensors (Basel).

[8] Xie B (2011). Effects of an eHealth literacy intervention for older adults. J Med Internet Res.

[9] Charness N, Boissy P, Demiris G, Krupinski EA, Lai AM, Lopez AM (2010). How human factors can influence the elderly in the use of telemedicine. Telemed J E Health.

[10] Young R, Willis E, Cameron G, Geana M (2014). "Willing but unwilling": attitudinal barriers to adoption of home-based health information technology among older adults. Health Informatics J.

[11] Davis FD (1989). Perceived usefulness, perceived ease of use, and user acceptance of information technology. MIS Q.

[12] Venkatesh V, Morris MG, Davis GB, Davis FD (2003). User acceptance of information technology: toward a unified view. MIS Q.

[13] Alsswey A, Al-Samarraie H (2019). Elderly users’ acceptance of mHealth user interface (UI) design-based culture: the moderator role of age. J Multimodal User Interfaces.

[14] van Houwelingen CT, Ettema RG, Antonietti MG, Kort HS (2018). Understanding older people's readiness for receiving telehealth: mixed-method study. J Med Internet Res.

[15] Zhou M, Zhao L, Kong N, Campy KS, Qu S, Wang S (2019). Factors influencing behavior intentions to telehealth by Chinese elderly: an extended TAM model. Int J Med Inform.

[16] Cimperman M, Makovec BM, Trkman P (2016). Analyzing older users' home telehealth services acceptance behavior-applying an Extended UTAUT model. Int J Med Inform.

[17] Guyatt G, Oxman AD, Akl EA, Kunz R, Vist G, Brozek J, Norris S, Falck-Ytter Y, Glasziou P, DeBeer H, Jaeschke R, Rind D, Meerpohl J, Dahm P, Schünemann HJ (2011). GRADE guidelines: 1. Introduction-GRADE evidence profiles and summary of findings tables. J Clin Epidemiol.

[18] Kavandi H, Jaana M (2020). Factors that affect health information technology adoption by seniors: a systematic review. Health Soc Care Community.

[19] Tague N (2005). The Quality Toolbox. 2nd Ed.

[20] Jaana M, Sherrard H, Paré G (2019). A prospective evaluation of telemonitoring use by seniors with chronic heart failure: adoption, self-care, and empowerment. Health Informatics J.

[21] Mercer K, Giangregorio L, Schneider E, Chilana P, Li M, Grindrod K (2016). Acceptance of commercially available wearable activity trackers among adults aged over 50 and with chronic illness: a mixed-methods evaluation. JMIR Mhealth Uhealth.

[22] Lang C, Voigt K, Neumann R, Bergmann A, Holthoff-Detto V (2020). Adherence and acceptance of a home-based telemonitoring application used by multi-morbid patients aged 65 years and older. J Telemed Telecare.

[23] Zettel-Watson L, Tsukerman D (2016). Adoption of online health management tools among healthy older adults: an exploratory study. Health Informatics J.

[24] Currie M, Philip LJ, Roberts A (2015). Attitudes towards the use and acceptance of eHealth technologies: a case study of older adults living with chronic pain and implications for rural healthcare. BMC Health Serv Res.

[25] Pywell J, Vijaykumar S, Dodd A, Coventry L (2020). Barriers to older adults' uptake of mobile-based mental health interventions. Digit Health.

[26] Kim S, Choudhury A (2020). Comparison of older and younger adults' attitudes toward the adoption and use of activity trackers. JMIR Mhealth Uhealth.

[27] De Veer AJ, Peeters JM, Brabers AE, Schellevis FG, Rademakers JJ, Francke AL (2015). Determinants of the intention to use e-Health by community dwelling older people. BMC Health Serv Res.

[28] Herrmann M, Boehme P, Hansen A, Jansson K, Rebacz P, Ehlers JP, Mondritzki T, Truebel H (2020). Digital competencies and attitudes toward digital adherence solutions among elderly patients treated with novel anticoagulants: qualitative study. J Med Internet Res.

[29] Cao Y, Li J, Qin X, Hu B (2020). Examining the effect of overload on the mHealth application resistance behavior of elderly users: an SOR perspective. Int J Environ Res Public Health.

[30] Vaziri DD, Giannouli E, Frisiello A, Kaartinen N, Wieching R, Schreiber D, Wulf V (2019). Exploring influencing factors of technology use for active and healthy ageing support in older adults. Behav Inform Technol.

[31] Russell TG, Gillespie N, Hartley N, Theodoros D, Hill A, Gray L (2015). Exploring the predictors of home telehealth uptake by elderly Australian healthcare consumers. J Telemed Telecare.

[32] Cajita MI, Hodgson NA, Lam KW, Yoo S, Han H (2018). Facilitators of and barriers to mHealth adoption in older adults with heart failure. Comput Inform Nurs.

[33] Li L, Peng W, Kononova A, Bowen M, Cotten SR (2020). Factors associated with older adults' long-term use of wearable activity trackers. Telemed J E Health.

[34] Peeters JM, De Veer AJ, Van der Hoek L, Francke AL (2012). Factors influencing the adoption of home telecare by elderly or chronically ill people: a national survey. J Clin Nurs.

[35] Wade R, Cartwright C, Shaw K (2012). Factors relating to home telehealth acceptance and usage compliance. Risk Manag Healthc Policy.

[36] Li J, Ma Q, Chan AH, Man S (2019). Health monitoring through wearable technologies for older adults: smart wearables acceptance model. Appl Ergon.

[37] Askari M, Klaver NS, van Gestel TJ, van de Klundert J (2020). Intention to use medical apps among older adults in the Netherlands: cross-sectional study. J Med Internet Res.

[38] Cajita MI, Hodgson NA, Budhathoki C, Han H (2017). Intention to use mHealth in older adults with heart failure. J Cardiovasc Nurs.

[39] Quaosar GM, Hoque MR, Bao Y (2018). Investigating factors affecting elderly's intention to use m-Health services: an empirical study. Telemed J E Health.

[40] Lan Z, Liu H, Yang C, Liu X, Sorwar G (2020). Investigating influencing factors of chinese elderly users? Intention to adopt mHealth based on the UTAUT2 model. Proceedings of the Fourth International Conference on Biological Information and Biomedical Engineering.

[41] Meng F, Guo X, Peng Z, Lai K, Zhao X (2019). Investigating the adoption of mobile health services by elderly users: trust transfer model and survey study. JMIR Mhealth Uhealth.

[42] Parker SJ, Jessel S, Richardson JE, Reid MC (2013). Older adults are mobile too! Identifying the barriers and facilitators to older adults' use of mHealth for pain management. BMC Geriatr.

[43] Cimperman M, Brenčič MM, Trkman P, Stanonik MD (2013). Older adults' perceptions of home telehealth services. Telemed J E Health.

[44] Axelsson SW, Wikman AM (2016). Ready for eHealth. Older Swedes’ prceptions of eHealth services: using the PIADS Scale as a predictor for readiness. Technologies.

[45] Lin TT, Bautista JR, Core R (2020). Seniors and mobiles: a qualitative inquiry of mHealth adoption among Singapore seniors. Inform Health Soc Care.

[46] Charness N, Best R, Evans J (2016). Supportive home health care technology for older adults: attitudes and implementation. Gerontechnology.

[47] Tsai T, Lin W, Chang Y, Chang P, Lee M (2020). Technology anxiety and resistance to change behavioral study of a wearable cardiac warming system using an extended TAM for older adults. PLoS One.

[48] Rockmann R, Gewald H (2016). Technology-mediated health activities: an exploratory study on older adults. Proceedings of the 22nd Americas Conference on Information Systems (AMCIS).

[49] Buccoliero L, Bellio E (2014). The adoption of "silver" e-Health technologies: first hints on technology acceptance factors for elderly in Italy. Proceedings of the 8th International Conference on Theory and Practice of Electronic Governance.

[50] Best R, Souders DJ, Charness N, Mitzner TL, Rogers WA (2015). The role of health status in older adults' perceptions of the usefulness of eHealth technology. Hum Asp IT Aged Popul (2015).

[51] Knapova L, Klocek A, Elavsky S (2020). The role of psychological factors in older adults' readiness to use eHealth technology: cross-sectional questionnaire study. J Med Internet Res.

[52] Dolnicar V, Petrovcic A, Setinc M, Kosir I, Kavcic M (2017). Understanding acceptance factors for using e-care systemsdevices: insights from a mixed-method intervention study in Slovenia. Human Aspects of IT for the Aged Population.

[53] Hoque R, Sorwar G (2017). Understanding factors influencing the adoption of mHealth by the elderly: an extension of the UTAUT model. Int J Med Inform.

[54] Puri A, Kim B, Nguyen O, Stolee P, Tung J, Lee J (2017). User acceptance of wrist-worn activity trackers among community-dwelling older adults: mixed method study. JMIR Mhealth Uhealth.

[55] Ware P, Bartlett SJ, Paré G, Symeonidis I, Tannenbaum C, Bartlett G, Poissant L, Ahmed S (2017). Using eHealth technologies: interests, preferences, and concerns of older adults. Interact J Med Res.

[56] Diño MJ, de Guzman AB (2014). Using Partial Least Squares (PLS) in predicting behavioral intention for telehealth use among Filipino elderly. Edu Gerontol.

[57] Portz JD, Bayliss EA, Bull S, Boxer RS, Bekelman DB, Gleason K, Czaja S (2019). Using the technology acceptance model to explore user experience, intent to use, and use behavior of a patient portal among older adults with multiple chronic conditions: descriptive qualitative study. J Med Internet Res.

[58] Nymberg VM, Bolmsjö BB, Wolff M, Calling S, Gerward S, Sandberg M (2019). 'Having to learn this so late in our lives…' Swedish elderly patients' beliefs, experiences, attitudes and expectations of e-health in primary health care. Scand J Prim Health Care.

[59] Rooney J, Kubiak T, Westcott R, Reid R, Wagoner K, Pylipow P (2009). Building from the basics: Master these quality tolls and do your job better. Quality Engineering.

[60] Foster MV, Sethares KA (2014). Facilitators and barriers to the adoption of telehealth in older adults: an integrative review. Comput Inform Nurs.

[61] Whittemore R, Knafl K (2005). The integrative review: updated methodology. J Adv Nurs.

[62] Schulz R, Wahl H, Matthews JT, De Vito DA, Beach SR, Czaja SJ (2015). Advancing the aging and technology agenda in gerontology. Gerontologist.

[63] Peek ST, Wouters EJ, van Hoof J, Luijkx KG, Boeije HR, Vrijhoef HJ (2014). Factors influencing acceptance of technology for aging in place: a systematic review. Int J Med Inform.

[64] Maswadi K, Ghani NB, Hamid SB (2020). Systematic literature review of smart home monitoring technologies based on iot for the elderly. IEEE Access.

