# Supplementary material for: Design of an Integrated Acceptance Framework for Older Users and eHealth: Influential Factor Analysis
Source: J Med Internet Res. 2022 Jan 28;24(1):e31920. doi: 10.2196/31920 (PMC8838594; doi:10.2196/31920)
Supplement: Multimedia Appendix 1 [file jmir_v24i1e31920_app1.docx]

**Appendix 1.** Study overview and methods.

| Studies | Study method | Theory | Participants | | | Technology | Verification time |
| --- | --- | --- | --- | --- | --- | --- | --- |
|  |  |  | Number | Age (years), mean | Condition |  |  |
| Jaana et al [20] | - Longitudinal (6 months) - Quantitative | UTAUT^a^ | 23 | 75.2 | Heart failure | Telemonitoring | After installation/at the beginning of use |
| Mercer et al [21] | - One-off - Mixed | TAM^b^ | 32 | 64 | Chronic diseases | Activity trackers | After installation/at the beginning of use |
| Lang et al [22] | - Longitudinal (2 years) - Quantitative | —^c^ | 116 | 79.6 | Chronic diseases | Telemonitoring | Before installation and after installation/at the beginning of use |
| Zettel et al [23] | - One-off - Quantitative | — | 166 | 70.8 | Healthy | Web-based health management tools | Before installation (nonuser) and after enough use (over a month) |
| Cimperman et al [16] | - One-off - Quantitative | UTAUT | 400 | 61.13 | Healthy | Telehealth | Before installation |
| Currie et al [24] | - One-off - Mixed | — | 168 | 60-74^d^ | Chronic diseases | eHealth | Before installation (nonuser) and after enough use (regular user) |
| Pywell et al [25] | - One-off - Qualitative | Socioecological model | 10 | 68 | Depressive symptoms | mHealth^e^ | After installation/at the beginning of use |
| Kim and Choudhury [26] | - One-off - Qualitative | TAM | 30 | 71.5 | Healthy | Activity trackers | After enough use  (2 months-6 years) |
| De Veer et al [27] | - One-off - Quantitative | UTAUT | 1014 | 57-77^d^ | Healthy | eHealth | Before installation |
| Herrmann et al [28] | - One-off - Qualitative | — | 20 | 75 | Treated with anticoagulants | Digital adherence solutions | Before installation |
| Alsswey and Al-Samarraie [13] | - One-off - Quantitative | TAM | 81 | 60 | Healthy | mHealth | Before installation |
| Cao et al [29] | - One-off - Quantitative | — | 317 | 60 | Healthy | mHealth | After enough use  (mHealth regular user) |
| Vaziri et al [30] | - One-off - Mixed | TAM and social cognitive theory | 188 | 50 | Healthy | Telemonitoring | After enough use (> 2 months) |
| Russell et al [31] | - One-off - Quantitative | TAM | 306 | 50-68^d^ | Healthy | Telehealth | Before installation |
| Cajita et al [32] | - One-off - Qualitative | — | 10 | 66-83^d^ | Heart failure | mHealth | Before installation |
| Li et al [33] | - One-off - Quantitative | — | 214 | ≥65 | Healthy | Activity trackers | After enough use  (<6 mo and >6 months) |
| Zhou et al [15] | - One-off - Quantitative | TAM | 436 | ≥60 | Chronic diseases | Telehealth | Before installation |
| Peeters et al [34] | - One-off - Quantitative | Rogers’ diffusion of innovation theory | 254 | 77.8 | Chronic diseases | Telehealth | After enough use  (actual user of home telecare system) |
| Wade et al [35] | - Longitudinal (12 weeks or 24 weeks) - Quantitative | TAM | 61 | 80 | Chronic diseases | Telehealth | Before installation and after enough use  (3 months and 6 months) |
| Li et al [36] | - One-off - Quantitative | TAM | 146 | ≥60 | Healthy | Wearable health monitoring | Before installation |
| Askari et al [37] | - One-off - Quantitative | TAM | 364 | 75 | Healthy | mHealth | Before installation |
| Cajita et al [38] | - One-off - Quantitative | TAM | 129 | 71.3 | Heart failure | mHealth | Before installation |
| Quaosar et al [39] | - One-off - Quantitative | UTAUT | 245 | ≥60 | Healthy | mHealth | Before installation |
| Lan et al [40] | - One-off - Quantitative | UTAUT2 | 367 | ≥60 | Healthy | mHealth | Before installation |
| Meng et al [41] | - One-off - Quantitative | Trust transfer theory | 395 | ≥60 | Healthy | mHealth | Before installation |
| Parker et al [42] | - One-off - Qualitative | — | 41 | ≥60 | Chronic diseases | mHealth | Before installation |
| Cimperman et al [43] | - One-off - Qualitative | UTAUT and TAM | 87 | 55-75^d^ | Healthy | Telehealth | Before installation |
| Axelsson et al [44] | - One-off - Quantitative | — | 154 | 71.9 | Healthy | eHealth | Before installation |
| Lin et al [45] | - One-off - Qualitative | TAM | 35 | 67.8 | Healthy | mHealth | Before installation |
| Charness et al [46] | - Longitudinal (6 months) - Quantitative | — | 215 | 75.7, 71.7, and 71.8 (group) | Healthy and heart failure | Wearable health monitoring | After enough use (6 months) |
| Tsai et al [47] | - One-off - Quantitative | TAM | 112 | 50-99^d^ | Healthy and heart failure | Wearable health monitoring | Before installation |
| Rockmann and Gewald [48] | - One-off - Quantitative | TAM | 156 | 72 | Healthy | eHealth | Before installation |
| Buccolieroand Bellio [49] | - One-off - Quantitative | TAM | 109 | 72.2 | Healthy | eHealth | Before installation |
| Best et al [50] | - One-off - Quantitative | — | 194 | 74.1 | Healthy | eHealth | Before installation |
| Knapova et al [51] | - One-off - Quantitative | — | 250 | 66.14 | Healthy | eHealth | Before installation |
| Dolnicar et al [52] | - One-off - Mixed | — | 12 | 77 | Healthy | Telemonitoring | After enough use  (2 months) |
| Hoque et al [53] | - One-off - Quantitative | UTAUT | 274 | ≥60 | Healthy | mHealth | Before installation |
| van Houwelingen et al [14] | - One-off - Mixed | UTAUT | 256 | 70 | Healthy | Telehealth | Before installation (nonuser) and after enough use(<1 year and >1 year) |
| Puri et al [54] | - One-off - Mixed | TAM | 20 | 64 | Healthy | Activity trackers | After enough use  (42 days) |
| Ware et al [55] | - One-off - Qualitative | — | 15 | 67 | Healthy and chronic diseases | eHealth | After enough use (regular user) |
| Diño and de Guzman [56] | - One-off - Quantitative | UTAUT | 82 | 60-66^d^ | Healthy | Telehealth | After enough use (25-30 days) |
| Portz et al [57] | - One-off - Qualitative | TAM | 24 | 78.41 | Chronic diseases | eHealth | Before installation (nonuser) and after enough use (user: average 17 days) |
| Nymberg et al [58] | - One-off - Qualitative | — | 15 | 65-80^d^ | Chronic diseases | eHealth | Before installation |

^a^UTAUT: Unified Theory of Acceptance and Use of Technology.

^b^TAM: Technology Acceptance Model.

^c^Not applicable.

^d^Age presented as range instead of mean.

^e^mHealth: mobile health.
