# Supplementary figures and images for: Design of an Integrated Acceptance Framework for Older Users and eHealth: Influential Factor Analysis
Source: J Med Internet Res. 2022 Jan 28;24(1):e31920. doi: 10.2196/31920 (PMC8838594; doi:10.2196/31920)

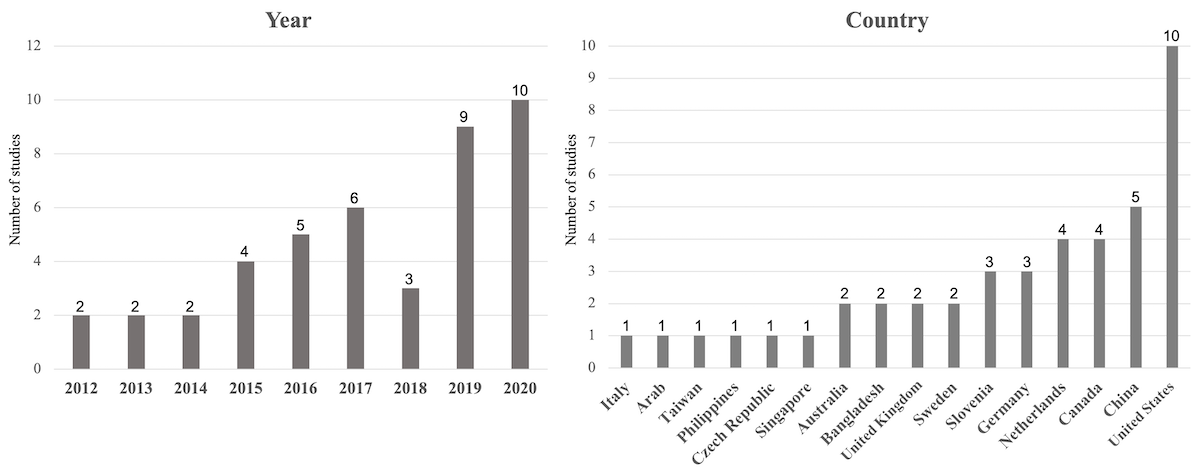

Supplement: Multimedia Appendix 2 [file jmir_v24i1e31920_app2.png]
